# Clinical research nurse and midwife as an integral member of the Trial Management Group (TMG): much more than a resource to manage and recruit patients

**DOI:** 10.1136/leader-2022-000641

**Published:** 2022-09-09

**Authors:** Clare Pye, Linda Tinkler, Mostafa Metwally

**Affiliations:** 1 Jessop Wing, Sheffield Teaching Hospitals NHS Foundation Trust, Sheffield, UK; 2 Freeman Hospital, Newcastle Upon Tyne Hospitals NHS Foundation Trust, Newcastle Upon Tyne, UK

**Keywords:** career development, clinical leadership, project management, research, nurse

## Abstract

**Background:**

The clinical research nurse/midwife (CRN/M) makes a unique contribution to research delivery in the National Health Service, resulting from a close therapeutic relationship with research participants. Investment in research infrastructure has led to nurses and midwives undertaking extended roles to deliver clinical research and evidence demonstrates the important contributions they make to the clinical research process, quality of research outcomes and most importantly the safe expert care of research participants. The value of the CRN/M’s contribution to the broader research team and acknowledgement of the importance of their input, however, remains unspecified and tacit in nature.

**Aim:**

To demonstrate the value a CRN/M has on overall trial design and performance when funded as a co-applicant and member of the Trial Management Group (TMG).

**Method:**

This briefing paper outlines the development and implementation of the CRN/M role and will describe its impact to promote the benefits of such a role as much more than a resource to recruit and manage participants.

**Results:**

Recognising CRN/Ms expertise, knowledge and contribution within this context is a positive step for the research agenda, individual career development and opportunity to introduce innovative ways of working to benefit the research landscape, ultimately contributing to the growth of the body of evidence available to influence patient care.

**Conclusion:**

When a CRN/M is funded as a co-applicant and member of the TMG, the role has a positive demonstrable impact on overall trial success.

## Background

 The clinical research nurse/midwife (CRN/M) makes a unique contribution to research delivery in the National Health Service (NHS), resulting from a close therapeutic relationship with research participants.^1^ Insight gained from such relationships has the potential to significantly benefit trial design, conduct and subsequent success. The utilisation and impact of this distinct insight is often an ad-hoc, unseen contribution, resulting from support offered to address trial recruitment challenges that is rarely resourced, or formally recognised as part of the Trial Management Group (TMG) or wider research process.^2^ However, when a CRN/M is funded as a coapplicant and member of the TMG, providing this expertise, knowledge and leadership, the role from the authors’ experience has a positive demonstrable impact on overall trial success.

Investment in research infrastructure has led to nurses and midwives undertaking extended roles to deliver clinical research^3^ and evidence demonstrates the important contributions they make to the clinical research process, quality of research outcomes and importantly the safe expert care of research participants.^4^ The value of the CRN/M’s contribution to the broader research team and acknowledgement of the importance of their input, however, remains unspecified and tacit in nature.

**Figure 1:**
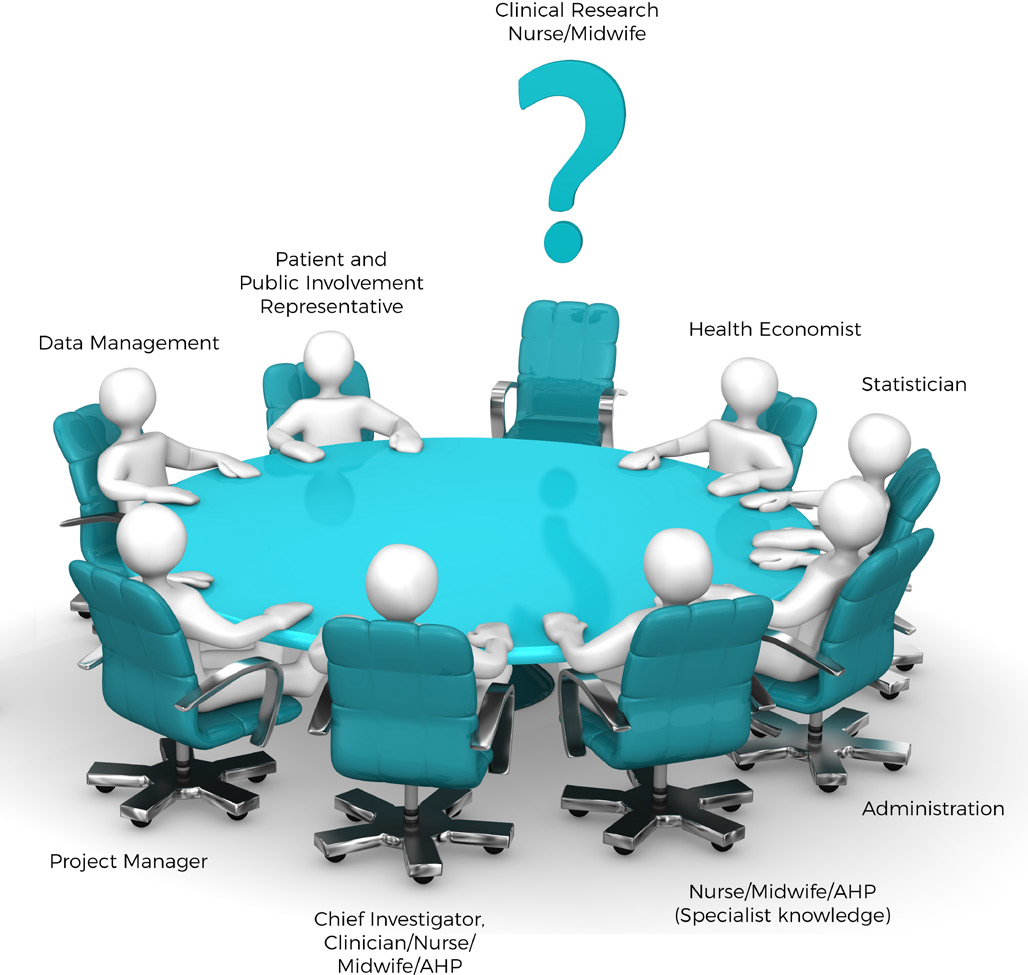
The clinical research nurse or midwife as an integral member of the Trial Management Group (TMG).

The innovative model described and depicted (figure 1) positions the CRN as an integral member of the Sheffield Centre for Reproductive Research (SCEPTR) as the role is embedded as a co-applicant and funded member of the team for all trials the SCEPTR team perform. This briefing paper outlines the development and implementation of this role and describes its impact to promote the benefits of such a role as much more than a resource to recruit and manage participants.

### Development

The model was developed and implemented by the SCEPTR CRN during the performance of The Endometrial Scratch,^5^ HELP Fertility? and STOP-OHSS trials and is informed by the authors’ experience of trials that often failed to consider potentially avoidable key issues having the potential to negatively influence participation for patients and research sites. These issues included:

- Restrictions resulting from unnecessarily tight inclusion/exclusion criteria.
- Conflicts between proposed research pathway and standard care pathways.
- Appropriate and meaningful Patient and Public Involvement (PPI).

Such factors can have significant consequences on performance, resulting in delayed or suboptimal recruitment, reduced opportunities for patients to participate, loss of funding and delays in obtaining evidence to influence practice^6^ and all ultimately impacting on health outcomes of the population.^7^ The CRN recognised this and advocated early preaward feasibility (during grant design phase) as a potential key element in addressing issues and was subsequently able to contribute significantly to the development of protocols and trial procedures.

### Implementation

This role within the TMGs evolved over time and is now fully embedded. Key responsibilities/activities undertaken as part of the role will be described in table 1.

**Table 1:** Examples of where the CRN/M can influence overall trial performance

Design phase—specific focus on potential patient research pathways and eligibility criteria	Influence on trial recruitment and performance
Knowledge of the clinical condition and experience in research delivery enables the CRN/M to contribute at all levels of discussion and decision-making but specifically to:Influence the proposed patient pathway…Collaborate with research networks/clinical colleagues/CRN/Ms etc…Support PPI members to develop skills and confidence…	To facilitate maximum involvement of participating research sitesTo ensure trial procedures are acceptable to potential participantsTo support the development of inclusionary eligibility criteriaTo influence proposed patient research pathways, recruitment strategies and patient facing documents
**Delivery Phase—specific focus on the clinical/research delivery aspects of the trial protocol**	**Influence on trial recruitmentandperformance**
Knowledge of the clinical condition, grant designed and experience in research delivery enables the CRN/M to provide clinical/research delivery expertise to all participating research sites in the support of trial performance but specifically to:The design and content of all patients facing documents prior to Patient and public involvement review/input… Ethical/governance approval… The design and content of trial database/case report form (CRF/eCRF) and data collection tools…Deputise for Chief Investigator…Resolve real-time eligibility issues…Facilitate accurate reporting of adverse events…	Good clinical research practice General trial delivery NHS research teams Strategies to facilitate recruitment Inexperienced CRN/Ms Factors within NHS organisations impacting on recruitment Ensures documents are acceptable to the population under investigation and relevant governing bodiesTo ensure only, the data required for the purposes of analysis will be collected and data collection tools are fit for use in the clinical settingTo support quality of data capturedProvides continuity and leadershipFacilitates recruitmentPromotes quality, accurate data collection and trial integrity

CRN/M, clinical research nurse/midwife; NHS, National Health Service.

### Design phase

Potential issues arising during trial delivery are avoided through CRN involvement in grant development and protocol design as the CRN works alongside the wider expertise of trial teams ensuring protocol and research pathways are optimal, not only to patients but also participating research sites. This is achieved through a range of activities.

The CRN’s knowledge of the proposed patient population informs the trial teams’ approach during grant design to identify and highlight where a proposed treatment regime may adversely affect availability of eligible patients. Through discussion across TMGs and participating sites, criteria are amended to be less restrictive while avoiding any negative influence on the primary outcome promoting inclusion of the population and contributing to the subsequent achievement of overall recruitment.

Factors limiting recruitment are common in trial performance; however, these are often not identified prior to recruitment commencing^8^ and the CRN’s role in influencing approaches to recruitment extends beyond contributions to inclusion/exclusion criteria during trial design. The SCEPTR CRN works within established research networks enjoying strong collaborative relationships, built over time, while gaining experience and skills to facilitate recruitment and therefore can positively influence proposed recruitment strategies.

The CRN/M, often the closest member of the research team to the participant, has a unique insight into how the condition under investigation affects the patient.^9^ The facilitation of meaningful PPI that is appropriately actioned can further positively impact trial outcome. PPI is provided by the Reproductive Health Research Public Advisory Panel—Sheffield Teaching Hospitals and importantly is led by the SCEPTR CRN, who overtime has supported the group to become confident, knowledgeable, and therefore able to actively influence trial design, including advocating for changes to outline funding proposals, enhancing trial materials and approach to patients.

### Delivery phase

The CRN’s specialist knowledge of the protocol, pathways and clinical condition under investigation enables this TMG member to deputise for the chief investigator where appropriate, influence recruitment, and provide pragmatic support to the design of the trial database and documentation. This ensures trial processes and procedures are fit for use in the clinical setting, aids real time resolution of eligibility and adverse event queries, all of which influence overall trial performance.

Due to insight and awareness of the role, the CRN can communicate, motivate and engage with NHS research teams using understanding and empathy, while being sensitive to conflicting pressures placed on individual research teams as well as actively facilitating the unblocking of issues within NHS organisations that cause delays, for example, different ideas and approaches to resourcing trials.

### Impact

The model described enables the instigation of early, pre-award feasibility and engagement with participating hospitals and has led to a range of positive impacts during the performance of our trials.

During the Endometrial Scratch (ES) trial,^5^ PPI articulated the need to amend follow-up procedures for participants resulting in a change to the outline proposal. While the TMG was initially concerned that such a notable change could negatively impact the funders’ decision, they recognised the modification was required. The funding decision was not negatively affected, and the application was successful, demonstrating the importance of a well-supported, well-led PPI group.

The CRN articulated the value of a network cluster approach to recruitment due to previous experience of trial delivery and with input from the Reproductive Health and Childbirth Research Network this recruitment strategy was built into the HELP Fertility? Trial and will be evaluated and considered for future recruitment strategies.

This TMG approach embeds full representation from a wide team of clinical and academic colleagues ensuring we limit, as much as possible, missing elements in trial design that might negatively influence performance and, with a funded seat firmly secured for a CRN to provide this input, the trial teams, to date have been 100% successful at safeguarding funding for all grants submitted.

## Discussion

The National Institute for Health Research CRN strategy highlights the need for visible leaders in clinical research nursing along with the imperative to demonstrate the impact the CRN/M has on the patient journey.^10^ Additionally, in November 2021, the chief nursing officer for England launched a strategic plan for research with an ambition to ‘create a people-centred research environment empowering nurses to lead, participate in, and deliver research, where research is fully embedded in practice and professional decision-making, for public benefit’.^11^

Typically, the expertise of the CRN/M supporting and addressing recruitment problems is regularly sought when recruitment fails to reach its target but by incorporating the experience of the CRN/M early in grant design, potential challenges with recruitment can be influenced helping prevent such issues once the trial is established.

TMG membership should comprise the right multidisciplinary members with the most appropriate skills and knowledge to positively influence aspects of trial performance from the outset. The inclusion of the CRN/M within the TMG is of equal importance and value to the expertise of a statistician or project manager, both of whom are integral to the constitution of a traditional TMG as we have found the CRN and project manager roles have developed into a successful collaboration, both acknowledging and influencing their individual contributions to trial performance.

In conclusion, when a CRN/M is funded as a co-applicant and member of the TMG the role has a positive demonstrable impact on overall trial success. However, it is important to acknowledge this paper is based on the authors’ opinions and aimed at generating discussion and debate about the role, its influence on trial performance as well as considering opportunities for CRN/M career development. The authors recognise there is not any empirical evidence to demonstrate this role has led to the improvements cited but anecdotally, based on our experience it does appear to be the case and therefore going forward suggest further research is performed to accurately measure its influence.

Recognising CRN/Ms expertise, knowledge and contribution within this context is a positive step for the research agenda and individual career development. The opportunity to develop skills and knowledge in clinical research, project management and trial methodologies as part of a TMG will serve to further develop CRN/M’s, benefitting academic development, career aspirations and opportunities. Finally, and perhaps most importantly introducing innovative ways of working to benefit the research landscape will ultimately contribute to the growth of the body of evidence available to influence patient care.
